# Earth to Mars: A Protocol for Characterizing Permafrost in the Context of Climate Change as an Analog for Extraplanetary Exploration

**DOI:** 10.1089/ast.2022.0155

**Published:** 2023-09-04

**Authors:** Kimberley R. Miner, Joseph Razzell Hollis, Charles E. Miller, Kyle Uckert, Thomas A. Douglas, Emily Cardarelli, Rachel Mackelprang

**Affiliations:** ^1^ Jet Propulsion Laboratory, California Institute of Technology, Pasadena, California, USA.; ^2^ The Natural History Museum, London, UK.; ^3^ US Army Corps of Engineers (CRREL), Washington, DC, USA.; ^4^ California State University at Northridge, California, USA.

**Keywords:** Permafrost, Cryosphere, Astrobiology, Earth Mars, Microbial life, Biogeochemistry

## Abstract

Permafrost is important from an exobiology and climate change perspective. It serves as an analog for extraplanetary exploration, and it threatens to emit globally significant amounts of greenhouse gases as it thaws due to climate change. Viable microbes survive in Earth's permafrost, slowly metabolizing and transforming organic matter through geologic time. Ancient permafrost microbial communities represent a crucial resource for gaining novel insights into survival strategies adopted by extremotolerant organisms in extraplanetary analogs. We present a proof-of-concept study on ∼22 Kya permafrost to determine the potential for coupling Raman and fluorescence biosignature detection technology from the NASA Mars Perseverance rover with microbial community characterization in frozen soils, which could be expanded to other Earth and off-Earth locations. Besides the well-known utility for biosignature detection and identification, our results indicate that spectral mapping of permafrost could be used to rapidly characterize organic carbon characteristics. Coupled with microbial community analyses, this method has the potential to enhance our understanding of carbon degradation and emissions in thawing permafrost. Further, spectroscopy can be accomplished *in situ* to mitigate sample transport challenges and in assessing and prioritizing frozen soils for further investigation. This method has broad-range applicability to understanding microbial communities and their associations with biosignatures and soil carbon and mineralogic characteristics relevant to climate science and astrobiology.

## 1. Introduction

Permafrost (perennially frozen rock, ice, soil, or organic material) covers approximately 24% of land in the Northern Hemisphere and stores 1700 billion tons of ancient carbon (Miner *et al.,* 2022). These dynamics are changing as the poles warm at nearly four times the global average on yearly timescales (Rantanen *et al.,* 2022). Permafrost warming in the Arctic is leading to both gradual and abrupt thaw, resulting in a ∼20% (3.6 × 10^6^ km^2^) increase of features exposing deep permafrost to the modern environment (Turetsky *et al.,* 2019). Previously frozen carbon is then vulnerable to the action of microbial communities who decompose it and release globally significant amounts of greenhouse gases into the atmosphere.

**FIG. 1.**
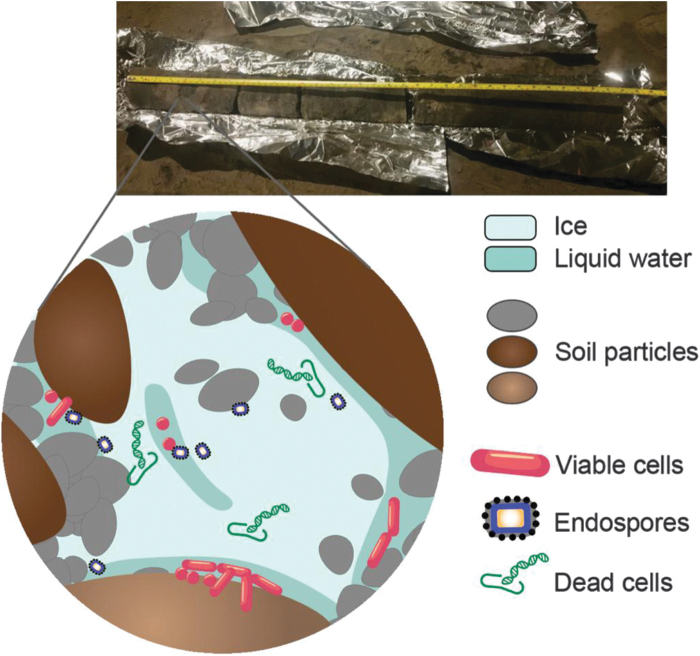
Permafrost core and representation of microbial communities in permafrost. The photograph is a permafrost core from the CRREL tunnel. The illustration represents active, dead, and dormant microbial cells. Viable cells persist in thin brine channels of liquid water. Endospores and dead cells, preserved by frozen conditions, are also found in permafrost.

Both gradual and abrupt thaw introduces novel stressors to permafrost ecosystems where microbial life persists in thin brine channels of liquid water, slowly metabolizing and transforming organic matter over millennia (Fig. 1) (Graham *et al.,* 2012; Ward *et al.,* 2017; Miner *et al.,* 2021). Gradual thaw describes a steady downward movement of near-surface permafrost. This occurs over broad spatial scales but does not always expose previously frozen soils to geomorphologic processes. Abrupt thaw is the formation of features like scars, slumps, and surface pockmarks. In both cases, prior to thaw the microbial communities have been sequestered away from the modern environment and show adaptations to the stressors associated with long-term survival in static subzero soils (Mackelprang *et al.,* 2017). The distribution of these communities varies over large spatial scales, but the complexity and composition of the soil matrix create microhabitats, which may house distinct microbial populations separated by centimeters (Jansson and Taş, 2014; Miner *et al.,* 2021). The extreme heterogeneity over multiple scales necessitates developing and applying rapid characterization techniques to determine the distribution and composition of soil microbial communities. Recent studies across regions have revealed substantial microbial diversity across extreme subzero environments (Zhang *et al.,* 2013; Jansson and Taş, 2014; Mackelprang *et al.,* 2017). The potential for microbes to act as both a lens into past ecosystems and a driver of modern ecological change makes it critical to polar science (Drake *et al.,* 2015; Chen *et al.,* 2020; Feng *et al.,* 2020; Miner *et al.,* 2021).

Off Earth, permafrost environments on icy planets and moons may hold some of the best opportunities for finding extraterrestrial microbial life (Smith and McKay, 2005; Douglas and Mellon, 2019; Bhartia *et al.,* 2021). While some analog spectra have been indexed for martian exploration (Razzell Hollis *et al.,* 2023), further characterizations of proxy microbial life are necessary. Microbial life in ancient permafrost represents hundreds to thousands of years of sequestration away from the modern environment and may present one of the best proxies available on Earth. Although Antarctic permafrost offers the best categorical analog for a martian low-carbon environment (Goordial *et al.,* 2016), these samples are difficult to access, retrieve, and process. For this first-order study, therefore, we sampled a ∼22 Kya permafrost core sample from the well-explored Cold Regions Research and Engineering Laboratory (CRREL) subsurface permafrost tunnel in Alaska. Future sampling efforts using this methodology will seek to incorporate samples that are more direct analogs to martian and other extraplanetary environments.

As a proof of concept, we apply the novel biosignature-detection fluorescence and Raman technology developed for the Mars Perseverance rover (Bhartia *et al.,* 2021; Razzell Hollis *et al.,* 2021; Uckert *et al.,* 2021) to identify and then characterize microbial community structure with DNA amplicon sequencing (Mackelprang *et al.,* 2017). Raman utilizes spectral mapping with a fluorescence spectrometer, where areas that fluoresce at certain magnitudes may indicate the presence of organic material (see methods below). Application of this combination of methodologies across permafrost environments will facilitate greater knowledge about the microbial communities, mineral and organic characteristics of permafrost, potential biosignatures, and how these dynamics are linked on geospatial scales. These cross-disciplinary perspectives aim to bring novel insights to enable the exploration of crucial and outstanding questions in both Earth and space sciences.

## 2. Materials and Methods

### 2.1. Sampling protocol

Permafrost cores were collected in May 2021 from the Cold Regions Research and Engineering Laboratory (CRREL) subsurface Permafrost Tunnel near Fairbanks, Alaska (64.951°N, 147.621°W). It is located along the valley floor of Goldstream Creek in a region of discontinuous permafrost. Permafrost in this area is syngenetic ice-rich silt (loess) formed through sediment deposition, causing the permafrost layer to expand upward (Kanevskiy *et al.,* 2016). This area also includes massive ice wedges and smaller ice features. Permafrost inside the tunnel is maintained at -3°C, which is similar to the mean annual air temperature of the Fairbanks area (Douglas and Mellon, 2019). The core was drilled at a diagonal where the tunnel wall meets the tunnel floor (20 m from the portal), dry-drilling using an 8 cm diameter SIPRE hand corer (Niendorf, 2019). We previously determined that permafrost from this portion of the tunnel is ∼19 Kya using radiocarbon dating (Mackelprang *et al.,* 2017). The core was drilled 1.2 m below the previously dated section. Given a sediment deposition rate of ∼0.5 mm per year (Johnson and Lorenz, 2000), we estimate our samples to be slightly older at approximately 22–23 Kya.

The protocols to handle and prepare the permafrost cores for analysis are well established (Mackelprang *et al.,* 2016, 2017; Burkert *et al.,* 2019), so here we summarize the salient details of the present study. The core was wrapped in sterile aluminum foil and transported to the laboratory in coolers with dry ice. Three subsections, two from the deepest part of the core (Deep 1 and Deep 2) and one from the middle of the core (Mid 1; Supplementary Fig. S1), were subsectioned in a room dedicated to processing permafrost samples, with a HEPA air purifier running continuously. Individuals processing the samples wore autoclaved lab coats, N95 masks, sterile nitrile gloves, hair coverings, and goggles. The outer ∼3 cm was cut away to remove the surface that contacted the corer using a wet tile saw (run without water) with a sliding table and autoclaved 10" continuous-rim diamond blades. The saw stage was covered with autoclaved aluminum foil. Saw blades, foil, and gloves were replaced frequently. Further subsectioning occurred using sterile knives within a laminar flow hood cleaned with 70% ethanol and exposed to UV light between samples.

### 2.2. Spectral mapping of permafrost

Due to the time required to complete microbial DNA sampling, fluorescence and Raman scans were run first. This allows for selective future sampling of microbial fluorescing regions *in situ* as initial fluorescence, and Raman scans can rapidly evaluate permafrost for entrained microbial structures in samples. Spectral mapping was performed using MOBIUS (Mineral and Organic Based Investigations using Ultraviolet Spectroscopy), a custom-built deep ultraviolet (DUV) Raman and fluorescence spectrometer designed by the NASA Jet Propulsion Laboratory. This same technology is employed on the Mars Perseverance rover. MOBIUS uses a 248.56 nm NeCu pulsed laser (Photon Systems, Inc.) (Uckert *et al.,* 2021) reflected off a 248 nm RazorEdge ultra-steep long-pass edge filter (Semrock, Inc.), focused onto the sample through a chromatically corrected *f*/4 objective lens (ThorLabs LMU-5x-UVB). The laser spot is annular in shape with an outer diameter of ∼44 mm and an effective illuminated area of 3540 μm^2^ (Razzell Hollis *et al.,* 2020, 2021). Raman scattering and fluorescence emission from permafrost molecules in the illuminated area was collected through the same lens and directed into a 550i spectrometer with a 250 mm slit width, spectrally dispersed by a holographic grating, and then recorded by a Horiba Symphony e2v 42-10 CCD liquid nitrogen cooled detector at -140°C. A minimum of 25 spectra at 100 μm spacing were measured for each strongly fluorescing region (hotspot) to ensure a representative average with a good signal-to-noise ratio (Razzell Hollis *et al.,* 2023). Identifying specific compounds depends on the number, position, and relative intensities of Raman peaks observed in the 800–4000 cm^−1^ range.

A holographic grating with 1800 lines/mm was used for Raman measurements, providing a spectral range of 800–4200 cm^−1^ (250–275 nm) at a spectral resolution of 3.8 cm^−1^/pixel. For fluorescence measurements, a grating of 300 lines/mm was used with a spectral range of 250–410 nm and a resolution of 0.16 nm/pixel. Spectral positions were calibrated before sample measurements by validating the position of the zero-order reflection, the secondary laser line at 252.93 nm (Bhartia *et al.,* 2021), and the Raman peaks of a known calibrant, acetonitrile. The laser was fired at 40 pulses per second during each measurement, with a pulse width of 40 μs. The pulse energy was limited to 1.8 μJ to minimize photochemical damage to any microbial cells in the sample.

Samples were trimmed to 10 × 10 cm to fit in a Linkam cryochamber for spectral mapping at -5°C. The cryochamber was actively cooled using pumped liquid nitrogen to maintain the physical and thermal consistency of the sample during measurements. Spectral mapping was achieved by moving the cryochamber and sample underneath the objective lens using a 3-axis motor-controlled stage, acquiring a spectrum at each point in a grid. Each sample was mapped using at least one large area fluorescence survey scan and multiple local Raman scans. Fluorescence survey scans covered at least 20 × 20 mm with 100 um point-to-point spacing for a minimum of 4000 individual point spectra, acquired using 25 laser pulses per point (0.625-second acquisitions). Local Raman maps covered 0.5 × 0.5 mm at 100 um spacing, resulting in 25 individual point spectra at 1200 laser pulses per point (30-second acquisitions).

Raman scan locations were selected based on the fluorescence intensity distribution obtained during the initial survey to prioritize areas of intense fluorescence that may indicate the presence of concentrated organic/biological material. A Canon camera provided context imaging of the mapped sample with a ∼20 mm/pixel resolution. Data processing was performed using custom Python programs and visualization tools based on a Loupe software package to process and visualize hyperspectral Raman and fluorescence data sets (Uckert, 2022). Spectral intensities were corrected to account for measured point-to-point variation in laser output. As previously published, the cosmic rays were automatically identified as outliers and removed along with the background signal (Uckert *et al.,* 2019). Raman peak positions, intensities, and widths were determined by fitting the spectrum with one or more Gaussian functions. Spectral maps were converted to false-color images to represent the intensity of each corresponding point spectrum at three different wavelengths associated with significant bands. False-color maps were also generated from each hyperspectral map's multivariate and cluster analyses. Each color band represents variance within spectra associated with variability in multiple spectral features' presence or intensity distribution.

### 2.3. DNA extraction, sequencing, and analysis

We performed triplicate DNA extractions from each core section (Mid 1, Deep 1, and Deep 2) directly from 0.5 g of soil using the FastDNA Spin Kit for Soil (MP Biomedicals, Santa Anna, CA, USA). An additional cleanup to remove humic material and other inhibitors was performed using the Qiagen DNeasy PowerClean Pro Cleanup Kit (Qiagen, Hilden, Germany). The 16S rRNA gene was amplified with the 515F/926R barcoded primer sets and conditions recommended by the Earth Microbiome Project. Libraries were sequenced on the Illumina MiSeq platform, generating 2 × 250 bp paired-end reads.

16S rRNA amplicon sequences were processed using the exact sequence variants (ESVs) pipeline in QIIME2, version 2022.2 (Bolyen *et al.,* 2019). Reads were demultiplexed, and quality filtered using the demux plugin, followed by the removal of chimeric sequences, dereplication, end-trimming, and read joining using DADA2 (Callahan *et al.,* 2016). We performed multiple sequence alignment using MAFFT (Katoh *et al.,* 2002) and generated phylogenetic trees with FastTree2 (Price *et al.,* 2010). We trained the feature classifier with RESCRIPt (Robeson *et al.,* 2021) against the SILVA SSU NR 99 138.1 database using the 515F/926R primer sequences (Quast *et al.,* 2013). Taxonomy was assigned to ESVs using the q2-feature-classifier plugin (Bokulich *et al.,* 2018).

Statistical analyses were performed within R (R Core Team, 2022), unless otherwise noted. We normalized counts using DESeq2 (Love *et al.,* 2014). Shannon alpha diversity index, weighted UniFrac distances, and Principle Coordinates Analysis (PCoA) were calculated within the phyloseq package (Mcmurdie and Holmes, 2013). Permutational Multivariate Analysis of Variance (PERMANOVA) and Analysis of Variance (ANOVA) tests were performed using the vegan package.

To identify close relatives of ESVs, we performed BLAST searches against the National Center for Biotechnology Information (NCBI) 16S ribosomal RNA sequences database (updated 2022/10/30) (Madden, 2013) and extracted sequences at a threshold of 97% sequence identity. Sequences were aligned using the SILVA Alignment, Classification, and Tree Service (Pruesse *et al.,* 2012). Phylogenetic trees were constructed using FastTree2 (Price *et al.,* 2010) and visualized using FigTree v1.4.4 (Suchard *et al.,* 2018).

## 3. Results

### 3.1. Raman and fluorescence results

**FIG. 2.**
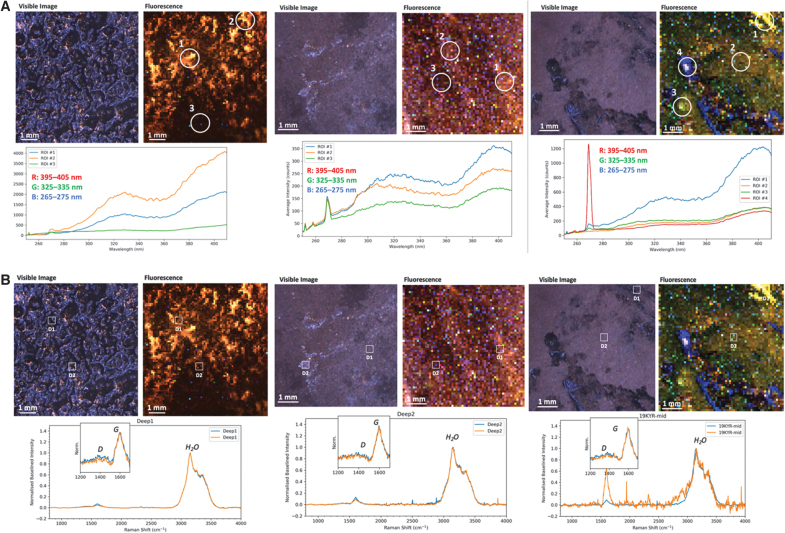
(**A**) Fluorescence and Raman survey scan results. Visible image, fluorescence, and Raman spectra for three samples at Deep 1, Deep 2, and Mid, respectively. (**B**) Fluorescence and Raman detail scan results. Visible image, fluorescence, and Raman spectra for three samples at Deep 1, Deep 2, and Mid, respectively.

The three permafrost samples exhibited consistent fluorescence signatures indicative of a similar continuum of organic matter present throughout each sample. This signature comprised broad fluorescence peaks at ∼320 and ∼410 nm, typical of biological macromolecules such as nucleic acids and proteins, which are consistent with expectations based on the prevalence of biomass in permafrost (Fig. 2A, 2B) (Fries and Steele, 2018). The two peaks were always observed together. They had a constant intensity ratio throughout each sample, suggesting that the local composition of organic material may not show statistical variation but does differ slightly between samples. Except for one sample, a much weaker, narrower peak was detected at 270 nm and assigned to the major Raman water peak (the OH stretching mode at 3200 cm^−1^). The appearance of this Raman peak in a fluorescence spectrum is consistent with the prevalence of water ice in permafrost (Fig. 2A, 2B). However, it was still relatively weak compared to fluorescence (Raman scattering cross-sections are typically 10^7^ times smaller per molecule than fluorescence). The relative intensity of the water band to organic fluorescence varied significantly between and throughout each sample and provides a measure of relative changes in the organic/ice concentration ratio.

**FIG. 3.**
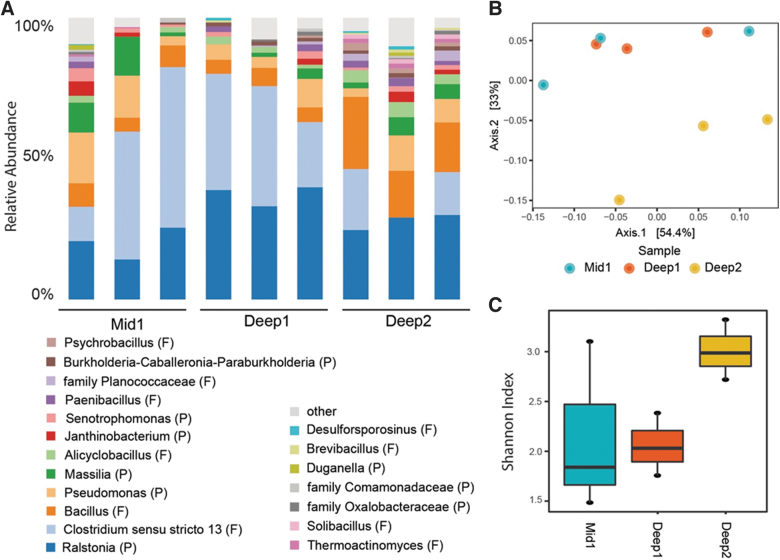
Genera, alpha and beta diversity of microbial communities in 19 Kya permafrost samples. (**A**) Relative abundance at the genus level. In cases where genus could not be determined, family is indicated. Phylum is indicated in parentheses after genus. P: Proteobacteria, F: Firmicutes. (**B**) PCoA ordination plot based on weighted UniFrac distances. (**C**) Box plot of the Shannon alpha diversity index.

Raman scans were relatively consistent between samples, exhibiting peaks assigned to either water ice or organic material. The dominant peak in every spectrum was the H_2_O stretching mode. However, when measured with higher spectral resolution and longer exposures, this was better resolved as an asymmetric peak with a narrow component at 3155 cm^−1^ and a broader, weaker component at 3350 cm^−1^ (Fig. 3A, 3B). The asymmetric bimodal shape is typical of the OH stretching mode in crystalline ice rather than liquid water.

Two additional peaks were observed at lower frequencies assigned to organic material: a strong, relatively narrow peak at 1598 cm^−1^ and a weaker, broader peak at 1350 cm^−1^, which were assigned to the G and D bands of carbonaceous organic material, respectively (Quirico *et al.,* 2009). Previous work has shown that variations in the positions, widths, and relative intensities of these two peaks can be diagnostic of the evolution of organic matter from amorphous, agglomerated carbonaceous material to highly ordered, pure carbon such as graphite (Schopf *et al.,* 2005; Quirico *et al.,* 2009; Delarue *et al.,* 2016). This process is driven by temperature and pressure, and the extent of organic degradation in permafrost may be used to infer the survivability of microbial biota.

The positions, intensities, and full-width-half-maxima of the D and G bands were reasonably consistent throughout the three samples, varying in intensity relative to the H_2_O peak. The relative strength of the D band, coupled with the broadness of the G band, is indicative of carbonaceous organic material that is poorly graphitized and only partially aromatized. Measured values are consistent with thermally immature organic material that has been somewhat degraded (*e.g.,* humic acid) (Quirico *et al.,* 2009). This is consistent with permafrost rich in decomposing biomass. While it is possible to detect and distinguish microbial cells by DUV Raman spectroscopy under highly controlled conditions (the microbial spectrum typically appears as a set of nucleic acid and protein peaks between 1100 and 1700 cm^−1^) (Sapers *et al.,* 2019), no distinct microbes could be conclusively detected in the permafrost samples. No mineral peaks were detected, suggesting ice and organic material are the major components of permafrost at this location.

### 3.2. 16S rRNA amplicon sequencing results

We performed 16S rRNA gene sequencing on nine samples (three replicates for each subsection: Mid 1, Deep 1, and Deep 2). Proteobacteria and Firmicutes were the most abundant phyla (48.7% and 48.3%, respectively), followed by Acidobacteria (1.1%) and Chloroflexi (0.62%) (Fig. 3A). We did not observe any methanogens. Ordinations indicated that section Deep 2 clustered away from Deep 1 and Mid 1 (Fig. 3B) but did not reach significance (PERMANOVA, P > 0.05). Similarly, alpha diversity was highest in section Deep 2 (Fig. 3C), but differences were not significant (ANOVA, P > 0.05). These data are consistent with other observations of similar patterns of microbial community structure in 19 Kya permafrost from the CRREL Permafrost Tunnel (Mackelprang *et al.,* 2017).

To better understand the microbial inhabitants of late Pleistocene permafrost, we compared ESV sequences of the five most abundant genera (*Clostridium sensu stricto 13, Bacillus, Pseudomonas*, *Massilia,* and *Ralstonia*), which together account for 78% of all sequences, to known 16S rRNA gene sequences. Close relatives (>97% sequence identity) have been isolated from an assortment of environments and conditions and employ a diversity of life and metabolic strategies, indicating that multiple divergent mechanisms can be employed to survive in ancient cryoenvironments.

**FIG. 4.**
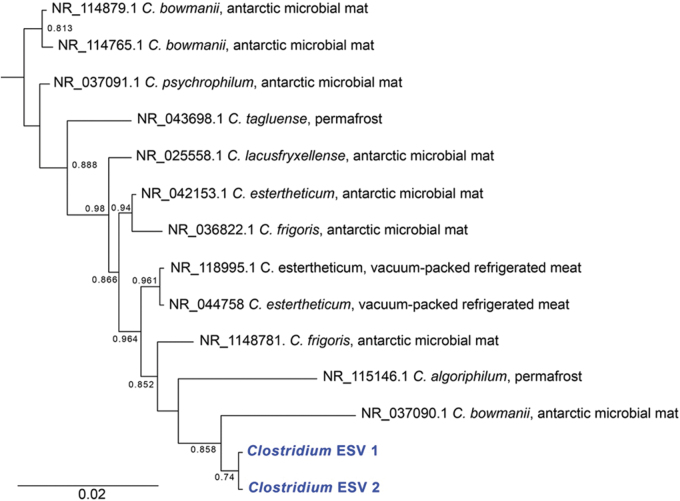
Phylogenetic tree of the 16S rRNA gene from *Clostridium* ESVs (in blue) and close relatives. For close relatives, the environment from which they were isolated is indicated after the name. The tree was constructed using FastTree 2.1. Local support values greater than 0.6 are indicated at the nodes. The tree was rooted using *Bacillus cereus* as an outgroup (Supplementary Figs. S2 and S3).

*Clostridium* and *Bacillus* are endospore-forming genera in the Firmicutes phylum. Twenty-three percent of all reads were from *Clostridium senso stricto 13*. Closely related taxa (>97% sequence identity, Fig. 4) had traits expected to be highly adaptive in an ancient permafrost environment. All are known to be psychrophilic or psychrotolerant and are saccharolytic anaerobes that gain energy through substrate-level phosphorylation, producing various fermentation end products (Spring *et al.,* 2003; Suetin *et al.,* 2009). Though they can form endospores, it is challenging to induce sporulation in several taxa (*C. bowmanii, C. psychrophilum,* and *C. lacusfryxellense*). In contrast to *Clostridium,* close relatives of *Bacillus* ESVs are primarily mesophilic (plus a few psychrophiles) and are derived from various environments and conditions. Previous data suggest that *Clostridium* may persist as vegetative cells in permafrost, while *Bacillus* form endospores (Burkert *et al.,* 2019).

*Pseudomonas, Ralstonia,* and *Massilia* are all genera from the phylum Proteobacteria. Taxa closely related to observed ESVs are primarily mesophiles and show enormous diversity and versatility (Supplementary Figs. S4, S5, S6). Members of the genus *Pseudomonas* make up almost 10% of the community. Bacteria in this genus are quintessential generalists, tolerating a wide range of habitats, temperatures, pH levels, and nutrient conditions (Lalucat *et al.,* 2020; Bell and Bell, 2021). Strains closely related to ESVs in our samples (>97% sequence identity) support this and have been derived from groundwater, wetlands, various temperate and cold soils, plants, and arthropod hosts. Close relatives of *Ralstonia* and *Massilia* ESVs are free-living in and widespread nature, occupying a variety of niches (Garrity *et al.,* 2005). At the same time, many of these same strains are opportunistic pathogens, demonstrating substantial phenotypic plasticity (Brown *et al.,* 2012).

## 4. Discussion

Permafrost cross-sections showed a widespread distribution of partially degraded organic carbon, pockets of water ice, and known microbial constituents. While we found no definitive spectral signatures corresponding to microbial community structure, this may be due to subsectioning methods. Given the high prevalence of organic material in permafrost, signatures from individual microbes may be lost in the background continuum of signals from all other organic matter present when analyzed with spectroscopy alone. To ameliorate this in our first-order analysis, core sections were divided into two pieces: one for spectroscopy and the other for microbial analysis. In the future, fluorescence and Raman spectroscopy can be applied to the core samples *in situ* to identify higher microbial community likelihood areas (demarcated by the minimal presence of ice or the higher presence of aromatic compounds), which can then be subsampled for DNA sequencing. Since there is fine-scale variation in permafrost soils, sequencing the same imaged regions will likely reveal relationships between spectral signatures and community structure.

Previous analyses from the tunnel demonstrated that replicate samples from directly adjacent cores had small variations in microbial community structure but highly significant differences when comparing permafrost of different radiocarbon ages located tens of meters apart (19, 27, and 33 Kya) (Mackelprang *et al.,* 2017). We also note that the communities from the samples we identified here (∼22 Kya) differ from these previously interrogated age categories. In the future, we plan to investigate spectral signatures corresponding to the different communities across chronosequences. Finally, while the spectroscopy for this study was completed using a stationary, lab-based instrument, the potential application of a field-based iteration of this tool would allow for rapid sampling without shipping or storage. Were this adaptation to the protocol to succeed, further large-scale integration of calibration curves would allow back-calculation of abundances in field maps to create the first record of microbial abundance and density in the Arctic.

### 4.1. The merits of Raman and fluorescence spectroscopy for permafrost investigations

Raman and fluorescence spectroscopies are highly sensitive to organic material and aromatic organic compounds, including DNA and protein (Bhartia *et al.,* 2012; Beegle *et al.,* 2015). DUV Raman exploits the combined signal enhancement of high-frequency excitation and molecular resonance with aromatic compounds to obtain greater sensitivity, enabling more rapid detection without altering the sample (Asher, 1993; Tarcea *et al.,* 2007; Razzell Hollis *et al.,* 2021). This is particularly valuable in searching for biosignatures, as aromatic compounds comprise a significant portion of biologically important macromolecules such as DNA, protein, and degraded biological materials such as energy substrates used in microbial metabolism (Nelson and Sperry, 1992; Wu *et al.,* 2001; Sapers *et al.,* 2019). Other Raman and fluorescence spectroscopy instruments can provide a similar index of spectral signatures, albeit with different signal yields and sensitivities depending on the laser wavelength and optics choice.

The strong UV absorption of aromatic compounds can lead to significant fluorescence yields under UV excitation, detectable at concentrations as low as 1 part per billion (Johnson *et al.,* 2011; Abbey *et al.,* 2017; Eshelman *et al.,* 2019). Any regions of intense fluorescence may indicate the presence of concentrated organic material and potentially microbes (Malaska *et al.,* 2020). Although some inorganic materials (minerals and rare earth elements) have been reported to fluoresce between 250 and 410 nm, inorganic fluorescence can typically be distinguished from organic fluorescence by narrow emission bands with significant spatial correlation to mineralogy (Shkolyar *et al.,* 2021).

### 4.2. Permafrost microbes on Earth

Extremotolerant microbes must employ strategies to maintain their structure, function, and metabolism at subzero temperatures. The taxa identified here suggest that multiple, divergent strategies can lead to survival in permafrost samples for tens of thousands of years. For example, the members of the *Clostridium* genus we identified are likely specialized for survival under cold anoxic conditions and likely persist as vegetative cells despite the ability to form endospores. In contrast, cells from the *Bacillus* genus are more likely than *Clostridium* to develop endospores as a survival mechanism (Burkert *et al.,* 2019).

Close relatives of *Pseudomonas, Massilia,* and *Ralstonia* ESVs are found in a remarkable diversity of conditions and environments, including spacecraft assembly rooms, soil, water, air, guts, and health clinics (Miller *et al.,* 2016; Nurjadi *et al.,* 2020; Narenkumar *et al.,* 2021). Most are mesophilic, though a few are known to be psychrotolerant. These taxa demonstrate substantial flexibility enabling them to adapt to and persist in diverse conditions, including permafrost. One survival strategy may be their well-described ability to form biofilms, which protect against environmental stressors and increase nutrient availability (Spring *et al.,* 2003; Adley *et al.,* 2005; Liu *et al.,* 2012; Nurjadi *et al.,* 2020; Al-ahmad *et al.,* 2021; Narenkumar *et al.,* 2021). Another is their metabolic diversity. For example, Pseudomonads closely related to the ESVs we identified can degrade sugars, amino acids, fatty acids, aromatics, and hydrocarbons (Verhille *et al.,* 1999; Stover *et al.,* 2000; Tvrzová *et al.,* 2006; Cámara *et al.,* 2007; Furmanczyk *et al.,* 2017; Gu *et al.,* 2020; Lalucat *et al.,* 2020; Zubkov *et al.,* 2021). This result is consistent with our spectroscopy-based observation that samples are rich in partially decomposed biomass.

Sequencing the 16S rRNA gene reveals bacterial and archaeal community structure but provides more limited information about permafrost microbial communities' functional potential and adaptative strategies. For example, the phylogenetic analysis of ESVs and closely related taxa presented here yields insights into the possible functions and adaptations of community members but needs to be confirmed using methods that more fully interrogate the community, such as metagenomics, cultivation, and so on. Here, the analysis of the microbial community is descriptive. Still, future applications of this protocol to include characterization of the microbial communities and expansion of permafrost ages and types will enable quantitative comparisons of communities and how they correspond to spectral data.

DNA-based studies do not distinguish between living, dead, and dormant cells. Previous studies suggest that approximately 50% of DNA from this location in the tunnel originates from dead cells; however, the same study also found that removing relic DNA did not substantially alter microbial community structure (Burkert *et al.,* 2019). This result is expected when the cell death and DNA degradation rates are similar across taxa. Therefore, we expect our results to represent the viable taxa at this location reasonably well.

Permafrost carbon characteristics are driven by the detrital plant material that accumulates during permafrost formation, variation in permafrost physicochemistry (*e.g.,* mineralogic soil composition, water content, temperature, pH), and the action of microbial communities as they slowly metabolize and transform organic matter through geologic time (Leewis *et al.,* 2020). Our study provides snapshots of carbon characteristics and microbial community structure in ∼20,000-year-old permafrost in Interior Alaska. Though there were no significant differences between core subsections, the microbial and spectrographic data indicate substantial heterogeneity. For example, the genus *Clostridium* ranges from 0% to 57% in relative abundance. In the future, combining both data types may introduce a novel means for determining if and how small-scale soil characteristics affect microbial spatial heterogeneity by extracting DNA from the precise regions scanned with MOBIUS technologies. Typically, physicochemical measurements are performed on “bulk” samples. Separate samples are used for microbial analyses. Here, it is possible to scan permafrost samples and then sequence the scanned regions yielding an unprecedented view of the covariation in soil and microbial characteristics.

These methods provide a unique means of studying permafrost in a changing climate. The 1700 billion tons of ancient carbon stored in permafrost exists in varying forms, from labile to recalcitrant (Jorgenson *et al.,* 2020) which can dramatically affect the mineralization rate during thaw (Drake *et al.,* 2015). Deployment of a mobile version of MOBIUS, similar to technology on the Perseverance rover, could rapidly evaluate the maturity and aromatization of permafrost carbon in regions vulnerable to thaw and track changes to carbon in regions of rapid (*e.g.,* thermokarst features) and slow (*e.g.,* top-down) thaw. Together with carbon dating of sampled strata, microbial analyses, and greenhouse gas measurements, this has the potential to identify and predict the relationships among many complex factors (*i.e.,* carbon and mineralogic composition, microbial communities, permafrost age, and thaw) and reveal how these ultimately drive the contribution of permafrost thaw to the climate change equation.

### 4.3. Exploring extraplanetary environments

Characterizing Earth's ancient permafrost microbial diversity and survival strategies provides a baseline to guide the search for microbial life on other frozen desert worlds. Permafrost is found in the subsurface of Mars and is expected in the mixed crustal and ice silicates of icy planets and moons across the Solar System, including Ceres, Callisto, and possibly Ganymede and Titan (Johnson, 2005; Damptz and Dombard, 2011; Dobiński, 2020). Microbial life in Earth's permafrost has survived for over a million years in some locations under extreme physical and environmental conditions (Johnson *et al.,* 2007; Morono *et al.,* 2020; Mühlemann *et al.,* 2020). These characteristics make them excellent proxies for understanding the potential for microbes to be viable in similar, or more extreme, environments on other worlds. For example, proxy methanogenic microbes in Earth's permafrost may explain the atmospheric methane seasonal cycle spikes observed in Gale Crater on Mars (Webster *et al.,* 2015, 2018).

The ability to rapidly and successfully characterize microbiologic and chemical conditions of ice and carbon-rich environments has important implications for extraplanetary exploration and studying permafrost on Earth. Recent breakthroughs in rapid DNA sequencing (Castro-Wallace *et al.,* 2017) may further enable exoplanet exploration. These methods could be combined with protocols for Raman on existing missions. For example, the ExoMars rover (European Space Agency, 2028) will use Raman to determine carbon-rich sites on Mars to prioritize sampling and characterization of organic carbon. While there is not currently a mission to Mars or any of the planetary moons that integrates rapid DNA sampling, these protocol overlaps could be explored in future decadal surveys.

Permafrost on Earth is dynamic in structure and formation, providing additional proxies for hypersaline environments such as brine lenses, legacy frozen substrate, and ice-rich sediment or ice wedges (Niederberger *et al.,* 2010; Bradley *et al.,* 2020). Much of Earth's permafrost is threatened by warming, which will change the chemistry and prevalence of unfrozen water in permafrost and surface water in areas overlying permafrost. The potential for microbial life to survive in conditions including high-salinity, low-temperature, or static cold environments can be explored on Earth in various permafrost locations (Gilichinsky *et al.,* 2003; MacKelprang *et al.,* 2011; Nikrad *et al.,* 2016). Many robust cryophiles among archaea and bacteria can grow and reproduce at temperatures down to -20°C, making them excellent analogs for martian biosignature carriers (Miteva *et al.,* 2009; Thurber *et al.,* 2020). As Mars sample return missions move forward, this protocol could also be applied to identify organic-rich core sections to prioritize for sampling and sequencing. New and novel analytical capabilities for analyzing the fractions of intact viable cells, intact dead cells, damaged cells, and biological debris will provide critical information about similar exoplanet environments. Using Earth as a benchmark, integrating *in situ* sampling methodologies with established microbial community interrogation techniques will save time in site exploration, retrieval methodology, and post-retrieval analysis while building a library to be used as a proxy for exoplanet biosignatures.

## 5. Conclusion

Up to 40% of northern latitude permafrost may thaw by the end of the century (Chadburn *et al.,* 2017), exposing billions of tons of organic carbon to microbial degradation. In light of this loss, the methods described here inform two crucial areas of research: the implications of thawing permafrost for climate change and enhancing life-detection strategies in extraterrestrial environments. This combination of spectroscopy and microbial community profiling tools will yield novel understanding of how relationships between biosignatures, carbon and mineral characteristics, and microbial communities develop through geologic time on Earth and other planets. For Earth science, the potential to test samples *in situ* could greatly reduce transport and laboratory cost, shortening the timeline from sampling to results. Creating an index of microbial community characteristics and survival mechanisms for extraplanetary exploration will inform the search for extraterrestrial life. As the search for life continues, any information to constrain search locations saves money and shortens the time to discovery. Proxies for exobiology detection currently exist on Earth, and every effort should be made to systematically characterize their diversity before they are lost to climate change.

## Supplementary Material

Supplemental data

Supplemental data

Supplemental data

Supplemental data

Supplemental data

Supplemental data

## Data Availability

Sequence data are available at the National Center for Biotechnology Information (NCBI) under BioProject accession number PRJNA909763.

## Abbreviations Used

ANOVA: Analysis of Variance
CRREL: Cold Regions Research and Engineering Laboratory
DUV: deep ultraviolet
ESVs: exact sequence variants
MOBIUS: Mineral and Organic Based Investigations using Ultraviolet Spectroscopy
NCBI: National Center for Biotechnology Information
PCoA: Principle Coordinates Analysis
PERMANOVA: Permutational Multivariate Analysis of Variance

## Notes

Associate Editor: Don Cowan
