# Supplementary material for: Earth to Mars: A Protocol for Characterizing Permafrost in the Context of Climate Change as an Analog for Extraplanetary Exploration
Source: Astrobiology. 2023 Sep 4;23(9):1006–18. doi: 10.1089/ast.2022.0155 (PMC10510695; doi:10.1089/ast.2022.0155)
Supplement: Supplemental data [file Supp_FigS1.pdf]

Supplementary Figures

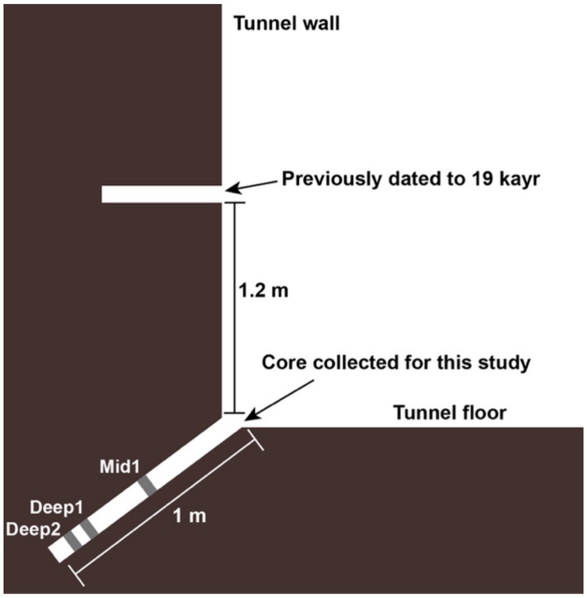

Supplementary Figure S1. Diagram of core location and subsections within the CRREL permafrost tunnel.
