# Supplementary material for: Earth to Mars: A Protocol for Characterizing Permafrost in the Context of Climate Change as an Analog for Extraplanetary Exploration
Source: Astrobiology. 2023 Sep 4;23(9):1006–18. doi: 10.1089/ast.2022.0155 (PMC10510695; doi:10.1089/ast.2022.0155)
Supplement: Supplemental data [file Supp_FigS2.pdf]

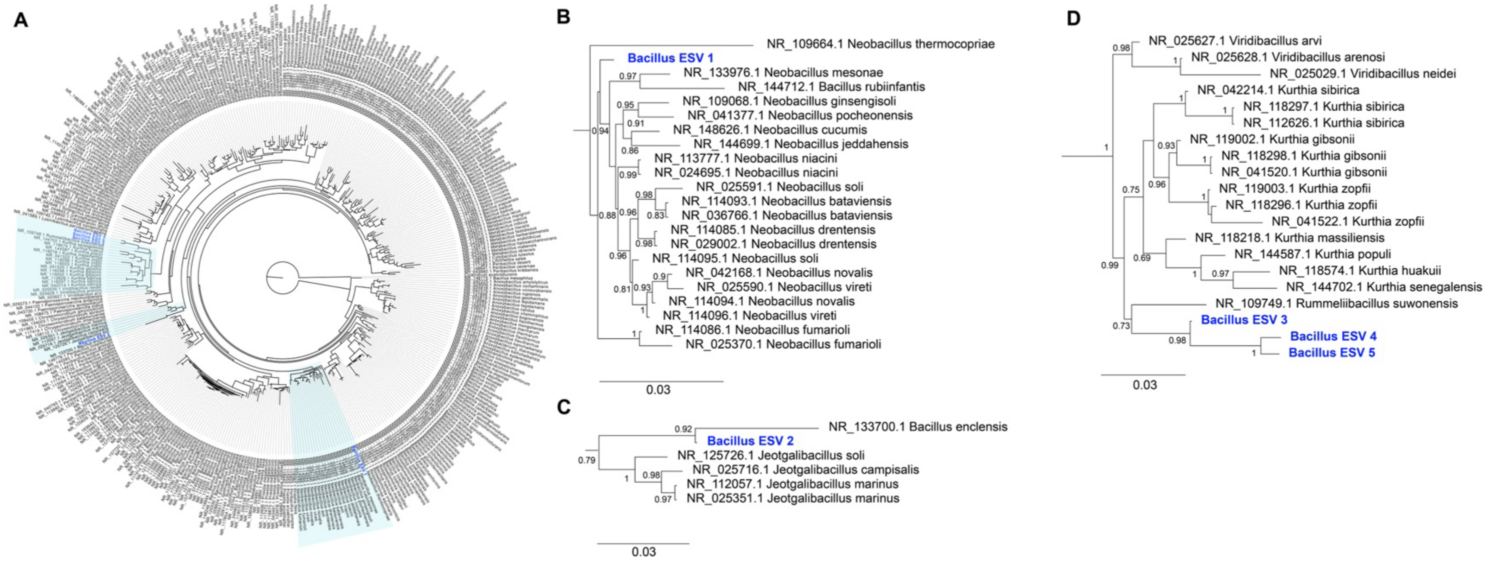

Supplementary Figure S2. Phylogenetic trees of the 16S rRNA gene from *Bacillus* ESVs and close relatives. Because of the large number of sequences matching *Bacillus* ESVs at 97% sequence identity, the trees were split into Supplemental Figure 2 (this figure) and Supplemental Figure 3. Clades with ESVs are indicated by a blue highlight in panel A and at an increased scale in panels B, C, and D. Local support values greater than 0.6 are indicated at the nodes. The tree was rooted using *Clostridium acetireducens* as an outgroup. The full high-resolution phylogenetic tree is on FigShare. Temporary link for review is https://figshare.com/s/98328332cd61873ec38d. DOI will become active after publication. 10.6084/m9.figshare.21699449
