# Supplementary material for: Earth to Mars: A Protocol for Characterizing Permafrost in the Context of Climate Change as an Analog for Extraplanetary Exploration
Source: Astrobiology. 2023 Sep 4;23(9):1006–18. doi: 10.1089/ast.2022.0155 (PMC10510695; doi:10.1089/ast.2022.0155)
Supplement: Supplemental data [file Supp_FigS6.pdf]

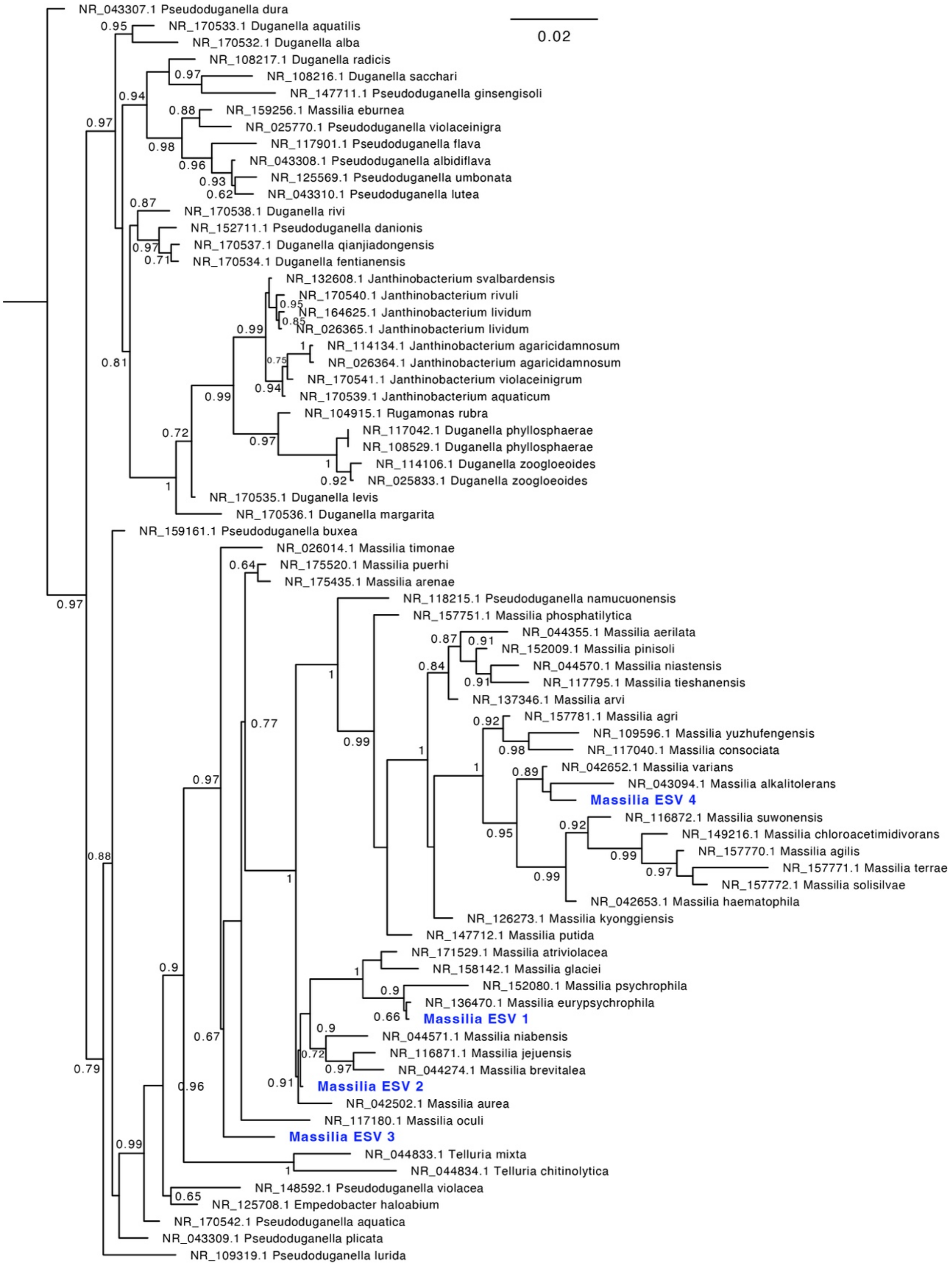


**Supplementary Figure S6.** Phylogenetic trees of the 16S rRNA gene from *Massilia* ESVs and close relatives. Local support values greater than 0.6 are indicated at the nodes. The tree was rooted using *Comamonas sp. MBIC3885* as an outgroup (not shown).
